# Child responsible personnel in adult mental health services

**DOI:** 10.1186/s13033-016-0098-y

**Published:** 2016-10-05

**Authors:** Camilla Lauritzen, Charlotte Reedtz

**Affiliations:** Regional Centre for Child and Youth Mental Health and Child Welfare, RKBU-Nord, UiT-Arctic University of Norway, 9037 Tromsø, Norway

**Keywords:** Systems change, Parental mental illness, Implementation, Child responsible personnel, Adult mental health services

## Abstract

**Background:**

Children who have parents with mental health problems are a vulnerable group. Intervening early to support parents with a mental illness can contribute to improve outcomes for children. Rigging the adult mental health system in such a manner that child responsible personnel are designated in wards is a strategy to systematically address the needs of families. It has since 2010 been mandatory for Norwegian hospitals to appoint such personnel in all hospital wards. The current study aimed to investigate the appointment of child responsible personnel in the adult mental health services in a regional hospital with local clinics. Additionally, to describe the characteristics of child responsible staff in terms of gender and educational background, their competence, clinical practice and knowledge about parental mental illness. A final aim was to study whether or not the clinics had established collaboration with other services concerning follow-up for the children of parents with mental illness.

**Method:**

Participants in this study are the staff at psychiatric clinics in a large university hospital in Norway. Practitioners were asked to answer a questionnaire prior to the initial process of implementing the new legislation in 2010 (N = 219). After a three-year period of implementing routines to adopt the new law in the clinic, the same survey was sent out to the staff in 2013 (N = 185) to monitor if changes were taking place. To study if the changes were sustained within the clinics, we conducted a two-year follow up in 2015 (N = 108).

**Results:**

The results indicated that the systematic work to change clinical practice in the participating hospital had made a difference. Routines to follow up children’s patients after the new legislation had to some extent been implemented. The child responsible personnel had more knowledge and awareness about the consequences of parental mental illness for children.

**Conclusion:**

The results of this study suggested that the systems change of establishing child responsible personnel within adult mental health services may be a tool contributing to safeguarding children of mentally ill parents. However, the role of being a child responsible should be further developed and defined.

## Background

Several studies the past decade have documented that children who have parents with mental health problems, are an exposed and vulnerable group [1–3]. The knowledge base about the risk factors related to having parents who are mentally ill is growing, and there is evidence to support the fact that these children run higher risks of a number of problems, e.g., family conflicts, poor parenting, abuse and neglect [4, 5]. There is also a significant risk of these children developing serious and long-lasting mental health problems themselves [6, 7]. Research has documented a higher risk of several mental health problems in the offspring of parents with mental health problems, i.e., depression, anxiety disorders, substance abuse, eating disorders and personality disorders [8–10].

In Norway, 37.3 % of all children have one or both ‘parents experiencing mental health problems, according to estimates by the Norwegian Public Health Institute [11]. Other Norwegian studies estimated that one-third of patients in outpatient clinics had custody and responsibilities for minor children [12, 13]. The findings in these studies are in line with international research [14–16].

Targeting adverse influences on children and parents, and intervening early may improve outcomes for children [7, 17]. Early interventions that support parents with a mental illness and their children can mitigate vulnerabilities and thereby contribute to the positive development of the next generation [18, 19].

However, before 2010, there were no formalized systems in the Norwegian mental health services for adults to record whether patients had children or not. Thus, many of these children remained undetected by health services and other relevant agencies [20]. The lack of procedures to identify the children living in families affected by parental mental illness, made provision of support for the families difficult. The awareness about the inadequate system surrounding these families has been growing among professionals in the field, researchers and the Norwegian government [20]. The adult mental health services is a central arena in order to detect children who have mentally ill parents, and formal procedures to protect children in cases where parents are receiving treatment for mental health problems was therefore facilitated [21].

Several modifications were made to two different acts within the Norwegian health legislation in order to improve the situation for these families. These acts are the Health Personnel Act [22] and the Specialized Health Services Act [23]. The new legislation became effective in 2010. The modified Health Personnel Act (§ 10 a) made it mandatory for all health professionals to (1) identify if patients have children and (2) to provide information and necessary follow-up for children under 18 years who have parents that receive health care for mental illness, substance abuse disorders or serious somatic illness or injury. Health care personnel are obligated to carry out conversations with patients who are parents of minor children, about the children’s need for information and follow-up. A crucial instrument for health personnel in order to change the clinical practice related to patients minor children, was an amendment which made it mandatory for all hospitals to appoint *child responsible personnel* in wards, clinics and institutions [21]. The intention was that child responsible personnel in the hospitals would be responsible for promoting and coordinating support for the children of patients. The legislation is not very specific on how individuals are designated as child responsible personnel within the clinics. The law only states that all clinics and wards within clinics are to have designated child responsible staff. They are designated this task on top of their regular duties and roles at work, and there are no specific criteria describing who should be selected. According to the Specialized Health Services Act, the core function of the child responsible personnel are to systematically address the needs of families, keep health professionals in their clinics updated about patients children and to promote the interests of the children [21].

However, previous studies have shown that it is difficult to implement practice changes in the adult mental health services [24]. A limitation with the new Specialized Health Services Act is that it does not specify what tasks, skills, competence and knowledge child responsible personnel need, and the role and authority of the child responsible personnel is therefore unclear. Existing literature on practice change suggests that an approach that does not incorporate specific tools or descriptions of a specific intervention lacks the “mechanism” to change practice [25]. Incorporating new guidelines alone do not necessarily mean that practice will change [26]. According to implementation theory, an approach to successfully change any practice must also provide content and tools to practitioners so that new procedures and processes have functional components for change [25, 26].

One of the initiatives the legislation intended the child responsible personnel to take was to establish collaboration about the children of patients with other relevant agencies such as the child protection services or schools. Efforts to collaborate more with municipal services may serve as an outcome of increased attention and promotion of the interests of the children of mentally ill patients in the psychiatric clinic. The intention with this is also to strengthen the identification of children at risk and providing adequate services in the municipalities where the family of mentally ill patients live.

Until now, little is known about the tasks, competence, skills and knowledge of the child responsible personnel in Norwegian hospitals. We also lack knowledge about their professional background, and if they are doing anything different in the day-to-day practice within the services compared to other health personnel. The current study was set up to investigate these issues.

## Methods

### Aims of the study

The aims of the previous study were to (1) investigate whether or not the participating psychiatric clinics in a large university hospital in Northern Norway had established child responsible personnel according to the altered legislation, (2) to describe the child responsible personnel in terms of gender and educational background, (3) to investigate child responsible personnel’s competence and clinical practice as compared with the general staff, and (4) to describe child responsible staff’s knowledge in terms of the new legislation, child development, and consequences of parental mental illness, as compared to the general staff. By the term general staff, we refer to all staff of the psychiatric services that are not designated child responsible personnel. We were interested to detect if there were any changes in the knowledge base over three different time points (2010, 2013 and 2015) and if there were significant differences between the child responsible personnel and the rest of the staff. The final aim was (5) to study whether or not the clinics had established collaboration with other services concerning follow-up of some kind for the children of the parents with mental illness after the adoption of the new law.

### Participants and procedure

Participants in this study are the staff at psychiatric clinics in a large university hospital in Northern Norway. The university hospital consists of several decentralized clinics throughout the region. There were 16 outpatient and inpatient clinics participating, serving a large geographical area of 31 municipalities. The total workforce was asked to answer a questionnaire at three different time points. The first wave of data was collected prior to the initial process of implementing the new legislation in 2010. The recruitment for participation was done by e-mail. A total of 219 members of the staff responded, representing a response rate of 50 %. The respondents were 76 % women, and the majority was between 30 and 50 years old. After a three-year period of implementing routines to adopt the new law in the services investigated [27, 28], the same questionnaire was sent out to all staff, including the child responsible personnel in 2013 to monitor if changes were taking place. A total of 185 individuals responded at post-test, representing a response rate of 40.5 %. The respondents were 67 % women, and the majority was between 30 and 50 years old. To study if the changes were sustained within the clinics, we did a two-year follow up in 2015. A total of 108 members of the staff responded at two-year follow up, and the majority of the respondents were women (74 %). A total of 53.6 % were between 30 and 50 years old. The response rate at two-year follow up was 24.5 %. The proportion of female respondents were unchanged from pre-test to post-test (Chi square = 3.81) and from post to follow-up (Chi square = 1.60).

### Measures

#### Establishment of child responsible personnel

Two items were included to investigate the establishment of child responsible personnel. The first was whether or not the clinics had designated child responsible personnel, i.e. “Have personnel at your clinic been designated as child responsible personnel?” The item was answered yes/no. The second was if personnel regarded that there were sufficient child responsible personnel designated at their clinic.

#### Demographic and work characteristics

Personal demographic variables included age, gender and education.

#### Child responsible personnel’s competence and clinical practice

To evaluate the perceived competence and clinical practice of the child responsible staff, nine items were included. The items included questions about the actual tasks of the child responsible staff, e.g., “In my clinic the child responsible staff have developed routines to safeguard the children of patients”. Additionally, items tapped into the perceived competence of the child responsible staff, e.g., “The child responsible staff at my clinic have the necessary competence to promote and coordinate follow-up on the patients’ children”. The items were answered on a five-point Likert-scale from “to a very large extent” (5) to “to a very little extent” (1). The computed Cronbach’s Alpha for tasks and competence was 0.91.

#### Health personnel’s knowledge

We assessed knowledge about children of mentally ill patients in the total staff using the *Family focused mental health practice questionnaire* [29]. The questionnaire was adapted to the Norwegian context with permission of the authors. We included nine items to assess the knowledge of the Child responsible staff as well as the general staff. Sample questions are: “To what extent would you say you have adequate competence to safeguard the children of your patients?” and “To what extent would you say you have knowledge about the consequences of mental illness for the offspring?” To study skills/knowledge about how to organize follow-up for children of patients, we asked: “To what extent do you have knowledge about how to develop and establish interventions for children of patients?” A sample question on the knowledge about the new legislation is: “To what extent do you have knowledge about the new legislation on the patients’ children?” The items were answered on a five point Likert-scale from “to a very large extent” (5) to “to a very little extent”. The Cronbach’s Alpha for the Knowledge subscale was 0.86.

#### Collaboration with other agencies to safeguard the children

To investigate if staff in the participating clinics had initiated a collaboration with other services regarding the children of patients we included seven items. A sample item is: “How would you describe the collaboration between the adult mental health services and the child protection services?” The items were answered on a five point Likert-scale from: “very good” (5) to “very poor” (1). “No existing collaboration” (0) was also an option. The Cronbach’s Alpha for the Collaboration subscale was 0.85.

### Data analysis

The data was exported from Quest Back to SPSS. All statistical analyses were performed with SPSS (Version 21). Descriptive analyses were used to explore the demographic details of the groups. Descriptive statistics was also used to study the competence and clinical practice of the child responsible personnel and the general staff. To investigate differences between these two groups we calculated means, and one-way ANOVA analyses were conducted to study development over time. Additionally, we performed a two tailed independent samples t test to detect differences between the child responsible personnel and the general staff in terms of follow up of patients’ children. Effect sizes were calculated using the Campbell Collaboration’s effect size calculator [30], and the effect size type chosen was Standardized Mean Difference (d). According to Cohen’s criteria, the standardized mean difference effect sizes should be interpreted as follows: 0.30 small effect, 0.50 medium effect and 0.80 large effect [31].

## Results

The results showed that the majority of the respondents worked at clinics in the participating hospital that had designated child responsible personnel at all three measurements. Some however, reported that such personnel had not been designated, even though the new legislation had made it mandatory. In 2010 (N = 219), 4.6 % reported they did not yet have child responsible personnel designated in their clinic. In 2013 (N = 185) and 2015 (N = 108) this number had increased to 5.9 %. This indicated that the focus on child responsible personnel had decreased to some extent since the new legislation was initiated in 2010. Respondents were also asked if they regarded the number of child responsible personnel in the clinic to be sufficient. In 2010, there were no significant differences between responses from child responsible personnel and the rest of the personnel, and both groups agreed that there were sufficient personnel designated as child responsible. In 2013, this had changed, and only child responsible personnel reported that there were sufficient amount of staff with child related tasks. However, this had declined again in 2015 to no significant differences.

The majority (75 %) of the child responsible personnel was between 41 and 60 years old at all measurement points. Most of the child responsible staff in our sample were women at all three time points; in 2010 100 % (N = 28), in 2013 90.5 % (N = 21) and in 2015 89 % (N = 9). In 2010 and in 2013 the largest group of child responsible personnel that responded to our survey were nurses. In 2015, the main group of responding child responsible personnel were environmental therapists. These are mainly staff with education at bachelor level, however they may differ in profession. An environmental therapist normally has a bachelor’s degree from a college or university, in e.g., social work, psychology, disability nursing or similar. Their responsibilities typically are participating in treatment teams and to facilitate social interaction or provide professional guidance to recipients of services in many areas.

For a detailed overview of the occupational backgrounds of the child responsible personnel see Table 1.

**Table 1:** Occupational background of child responsible personnel

Time	2010 (N = 28)	2013 (N = 23)	2015 (N = 9)
Occupation (%)			
Environmental therapist	17.9	21.7	55.6
Community worker	3.6	0	0
Medical doctor/psychiatrist	3.6	4.3	11.1
Nursing assistant	7.1	4.3	0
Psychologist	0	0	0
Nurse	32.1	30.4	0
Consultant	3.6	13.0	11.1
Disability nurse	10.7	8.7	11.1
Social workers	14.3	4.3	0
Physiotherapists, occupational therapist	3.6	0	0
Other	3.6	13.0	11.1

Results from evaluating the child responsible personnel’s competence and clinical practice, showed that there were no significant differences in how this personnel evaluated themselves and how they were evaluated by the general staff in 2010 and in 2015. At these two measurement points both the general personnel and the child responsible personnel reported that the competence, guidelines, provision of follow-up, interventions, initiated procedures, supervision, development of skills, and development of the organization were sufficient and well taken care of by child responsible personnel. In 2013 there were however significant differences on these variables (see Table 2), and child responsible personnel scored themselves higher on all variables, compared to how the general staff evaluated the child responsible personnel.

**Table 2:** Descriptive statistics and one-way ANOVA on the competence and clinical practice of the child responsible personnel and the general staff

	Time 1: 2010					Time 2: 2013					Time 3: 2015				
	CR (*N* = 28)		GS (*N* = 190)		ES	CR (*N* = 23)		GS (*N* = 156)		ES	CR (*N* = 9)		GS (*N* = 94)		ES
	M	SD	M	SD		M	SD	M	SD		M	SD	M	SD	
Competence	3.39	0.79	3.51	0.85	−0.14	4.04	0.46	3.54	0.78	0.67**	3.44	0.73	3.44	0.85	0.00
Guidelines	3.57	0.84	3.24	0.91	0.37	3.74	0.96	3.30	0.83	0.52*	3.89	0.60	3.27	0.89	0.71*
Providing follow-up	3.43	1.03	3.17	0.93	0.28	3.87	0.92	3.46	0.78	0.51*	3.89	0.33	3.37	0.79	0.68
Adequate interventions	3.11	0.83	2.84	0.96	0.29	3.35	0.89	3.00	0.79	0.44*	3.33	0.87	2.99	0.80	0.42
Initiated procedures	3.46	0.74	3.18	0.93	0.31	3.83	0.72	3.36	0.82	0.58**	3.56	0.73	3.17	0.89	0.44
Supervision	3.61	0.67	3.30	0.93	0.34	3.74	0.62	3.30	0.91	0.50*	3.56	0.53	3.10	1.08	0.44
Develop skills	3.74	0.66	3.63	0.75	0.15	4.00	0.52	3.61	0.76	0.53*	3.67	0.50	3.40	0.84	0.33
Develop organization	3.43	0.74	3.34	0.83	0.11	3.57	0.79	3.13	0.91	0.49*	3.33	0.87	2.93	0.97	0.42

*CR* child responsible, *GS* general staff, *ES* standardized mean difference (d)

* *p* < 0.05

** *p* < 0.01

*** *p* < 0.001

Child responsible personnel’s knowledge in terms of the new legislation, child development, and consequences of parental mental illness was assessed at all three time points. In 2010, the child responsible personnel scored significantly higher on knowledge about the new legislation as well as the amount of training they received than the general staff. There were no differences between this group and the general staff on the other variables. However, in 2013, the child responsible personnel also reported higher knowledge about parental mental illness and the consequences of this for the offspring. In 2015, the child responsible staff still reported higher knowledge about the legislation regulating the patients who are parents, compared to the general staff. However, the level of competence regarding child development was steadily rising among the general staff through all measurement points. Knowledge about parental mental illness was no longer significantly different between the two groups in 2015. For details on knowledge, see Table 3.

**Table 3:** Descriptive statistics and one-way ANOVA on the knowledge about parental mental illness, legislation and child development

	Time 1: 2010					Time 2: 2013					Time 3: 2015				
	CR (*N* = 28)		GS (*N* = 190)		ES	CR (*N* = 23)		CR (*N* = 156)		ES	GS (*N* = 9)		CR (*N* = 94)		ES
	M	SD	M	SD		M	SD	M	SD		M	SD	M	SD	
PMI parenting consequences	3.93	0.47	3.74	0.71	0.28	4.13	0.34	3.96	0.62	0.29	3.89	0.60	4.00	0.57	−0.19
Legislation knowledge	3.93	0.81	3.02	0.82	1.11***	3.87	0.69	3.14	0.83	0.90***	3.78	0.44	3.20	0.78	0.76*
Received training	3.43	0.98	2.33	0.96	1.14***	3.65	0.89	2.62	0.89	1.16***	3.67	0.71	2.47	1.03	1.19**
PMI offspring consequences	3.89	0.63	3.68	0.78	0.28	4.30	0.47	3.89	0.68	0.62**	3.89	0.60	4.02	0.57	−0.22
Child development	3.82	0.61	3.75	0.70	0.10	4.00	0.54	3.92	0.61	0.13	3.89	0.33	4.02	0.51	−0.26
Prevention	3.73	0.72	3.59	0.69	0.20	4.00	0.67	3.85	0.61	0.24	3.78	0.44	3.86	0.56	−0.15
Family focused experience	2.67	1.07	2.71	1.07	−0.04	3.09	0.85	2.82	0.96	0.29	3.22	0.97	2.75	1.04	0.45

*CR* child responsible, *GS* general staff, *PMI* parental mental Illness, *ES* standardized mean difference (d)

* *p* < 0.05

** *p* < 0.01

*** *p* < 0.001

To investigate if clinical practice had changed in relation to collaboration with other services, we asked all respondents how they perceived the quality of such collaboration. In 2010, the child responsible staff reported significantly higher contact with the child protection services and the public health nurses than the general staff did. In 2013, there were significant differences between the groups in terms of collaboration with the following services: child protection services, public health nurses in municipalities, child and adolescent mental health services and municipal educational psychology services. In 2015, the only significant differences between the groups in terms of collaboration with relevant agencies about the patients’ children, was with the educational psychology services and schools. For a detailed overview, see Table 4. Levene’s test was conducted to assess equal variances between the two groups at all three time points. Our data consistently violated the assumption of equal variance, and the information reported in Table 4 refers to the estimates based on *equal variances not assumed* in SPSS.

**Table 4:** Two-tailed independent samples t test child responsible and general staff in terms of initiated collaboration for follow-up of patients’ children

	Time 1: 2010					Time 2: 2013					Time 3: 2015				
	CR (*N* = 28)		GS (*N* = 190)		ES	CR (*N* = 23)		CR (*N* = 156)		ES	GS (*N* = 9)		CR (*N* = 94)		ES
	M	SD	M	SD		M	SD	M	SD		M	SD	M	SD	
Child protection service	3.73	0.63	3.13	0.79	0.78***	3.71	0.56	3.34	0.71	0.53**	3.56	0.53	3.19	0.67	0.56
Public health nurse	3.79	0.71	3.26	0.72	0.74**	3.82	0.50	3.30	0.68	0.79**	3.50	0.54	3.34	0.64	0.25
Child mental health service	3.25	0.58	3.33	0.76	−0.11	3.89	0.57	3.38	0.72	0.73**	3.67	0.82	3.36	0.66	0.46
Education psychology service	3.08	0.29	2.99	0.62	0.15	3.43	0.51	3.11	0.61	0.53*	3.00	0.00	3.13	0.38	−0.36*
Schools	3.07	0.27	3.07	0.68	−0.40	3.31	0.70	3.13	0.61	0.29	3.40	0.55	3.02	0.36	1.00*
Kindergartens	3.00	0.00	2.99	0.65	0.02	3.21	0.70	3.10	0.61	0.18	3.00	0.00	2.98	0.50	0.04

*CR* child responsible, *GS* general staff, *ES* standardized mean difference (d)

* *p* < 0.05

** *p* < 0.01

*** *p* < 0.001

## Discussion

The main aim of this study was to describe the tasks, competence, skills and knowledge of the child responsible personnel and to compare this group with the general staff on relevant variables. The workforce in the adult mental health services stated that child responsible personnel had been designated in the majority of the clinics, as the new health legislation requires. However, in 2015; 5 years after the law was changed, there were still about 6 % who reported that child responsible personnel had not been designated in their clinic. This finding may indicate that the Specialized Health Services Act were not fully implemented in the participating regional hospital. In 2010 all health personnel agreed that there were sufficient personnel designated as child responsible. However, in 2013, when scores on all knowledge variables and almost all competence variables were on their peak in the general staff, only child responsible personnel reported that there were sufficient amount of staff related to child tasks. In 2015, all health personnel again agreed that there were sufficient personnel designated as child responsible.

We were interested to see which gender and occupational background the child responsible personnel had. The finding that the large majority of child responsible personnel were female was interpreted to represent the reality in the clinics well. Our result is in line with findings from the Norwegian multicenter study [3], where 90 % of all the child responsible personnel were female. We expected that they would mainly be nurses or environmental therapists, based on previous qualitative studies in the clinic [32], as well as the previous mentioned multicenter study. This assumption was to some extent supported in the previous study. In 2010 and in 2013 almost one-third of the child responsible personnel in our sample were psychiatric nurses. However, in 2015, this had however changed dramatically. There were no responding nurses who were designated child responsible personnel in 2015, and the largest group of respondents to the survey this year was environmental therapists designated as child responsible personnel. This may indicate large turnover rates in the child responsible position. If the reason why so few psychiatric nurses responded to the survey in 2015 is related to extensive workload in the role as child responsible personnel, this gives reason for concern, as they may have discontinued their responsibilities for the same reason. In the existing body of literature on barriers to integrating a family focused practice in adult mental health settings [28, 33, 34], there are several likely explanations to why child responsible personnel may wear out in this role. Financial cutbacks, lack of time to plan work with patients and their children, reluctance among both staff and patients to be involved in family related work, limited supervision, limited skills training, as well as lack of streamlined procedures are common barriers to family focused practices [28, 33, 34]. These barriers are much the same as reported by the general staff in the participating hospital, pointing to the unclear and undefined responsibilities of being a child responsible [35]. Part of the intention behind the establishment of child responsible personnel was to become less dependent on devoted, informal and enthusiastic champions. Instead, the strategic move of appointing child responsible personnel was therefore intended to institutionalize efforts to make sure that children of all patients who are parents were safeguarded. If the turnover of child responsible personnel is very high, there are grounds to question if the establishment of child responsible personnel will address the needs of families systematically, or if the clinics still are dependent on informal champions. This needs to be explored in further research.

In terms of child responsible personnel’s competence and clinical practice concerning the children of patients, there were no significant differences between how this personnel evaluated themselves and how they were evaluated by the general staff in 2010. In 2013, the two groups were significantly different on all variables. However, in 2015 the differences between the groups were no longer present. This illustrates that in 2013, when the implementation process was at its peak, the child responsible personnel were evaluated as more competent compared to the rest of the staff. They were also considered active in providing guidelines and follow-up of personnel and families, as well as more active in providing procedures, interventions, supervision, and in the effort to develop skills, and the organization.

The results indicated that the systematic work to change clinical practice in the participating hospital actually did make a difference in terms of routines to follow up children’s patients in the first time period after the new legislation was enacted. This is also evident in an earlier study, were the general staff reported a significant improvement of the rates at which they assessed whether or not patients had children [28]. Nevertheless, the practice changes do not seem to have materialized to the extent that they are part of an institutionalized practice in the clinic in 2015. This may be due to sustainability issues. The goal with sustainability is the long term survival of newly implemented routines or system changes. Sustainability addresses the issue of how the new practice is to survive in the every day practice. According to the research literature on the sustainment of implemented practice changes, partial sustainability is very common [36]. This means that elements of the implemented practice may survive, but not necessarily all elements that make up a systems change package. We believe there are many challenges related to sustaining new clinical practice in mental health services related to children of mentally ill patients. First of all, the sustainability strategies should encompass strategic support within the organization. It is possible that the leaders of the mental health clinics within this clinic regarded their job done as they had designated the child responsible personnel. However, the success and sustainability of the child responsible function within the clinic requires substantial organizational support. The clinic has to retain an ongoing capacity for sustaining the interventions. Additionally, there must be ongoing recruitment of practitioners to carry out the interventions, which implies resource allocations. Sustaining interventions is reliant on core personnel bringing continuity to the work. Furthermore, any attempt to alter the mental health systems has to be properly anchored in the organization. The management must actively support the new practice, and this should be reflected in the policies within an organization, such as guidelines, service statements, protocols and interagency guidelines. Otherwise, the hopes of establishing the new routines within practice as usual is at risk.

Finances is also a big issue, as many attempts to change practice will fail to become sustainable because insufficient resources are provided. Cost-benefit analyses play an important role in the planning and decision making process, and sustainability issues need to be a part of the analyses. Perhaps if the child responsible had sufficient resources in terms of extra pay or sufficient time to ensure that the children of the patients are safeguarded, the role would perhaps not be afflicted with high turnover rates. Another solution could be to appoint only the therapists (i.e., the psychiatrists and/or the psychologists) as child responsible personnel, not the environmental therapists and the nurses who have different roles with the patient. The therapists are already in a treatment alliance with the patient, and should be in a position to initiate follow up of the patients’ children.

In terms of knowledge in terms of the new legislation, child development, and consequences of parental mental illness, the results showed that the level of knowledge among child responsible personnel was high throughout all three time points. This may indicate that the child responsible personnel did had a high awareness of the risk factors surrounding patients who are parents and their children, which was the intention behind the legislation. Furthermore, the level of competence among the general staff in the study was increased for these variables throughout all measurement points, and these personnel indicate that they have knowledge about parental mental illness and child development “to a large extent”. These findings may be explained by the work of child responsible personnel. In a multicenter study conducted in Norway [3], the results indicated that three out of four child responsible personnel contribute to keep the general staff updated. For the hospital to fully benefit from this knowledge, the role of being a child responsible should be given more real impact and influence in the day to day practice. If not, the discrepancy between practice and the existing knowledge about the negative impact of not including a child-focused practice will be maintained. The literature on this suggests that developing “champions” within organizations who can consistently advocate the process of change, and thereby contributing to workforce and organizational readiness to change, might be a sensible solution [37]. However, if the child responsible personnel are to attain such a role as champions and advocates for a family focused practice, they must be given adequate tools, time, resources and influence by the management and by the total workforce. Our experience from the present study is that using a standardized scale to explore changes in workforce knowledge and practice helps to interpret changes over the years. This is in line with other research pointing to the psychometric qualities of the family-focused mental health practice questionnaire, and especially organizational improvement to strengthen the quality of services to families affected with parental mental illness [38].

As for many of the other measured variables in this study, the peak of collaboration with other services was in 2013 for both child responsible personnel and the general staff. We were able to detect some differences between the groups in terms of collaboration, but at the same time there is a potential for development in this area for both groups. We know from previous research that the adult mental health services do not report concerns or initiate collaboration with child protection services to a large extent [39]. Child responsible personnel at all hospitals should be encouraged to collaborate and discuss cases with a variety of agencies in the local communities, e.g., schools, kindergartens, the pedagogical-psychological service available for schools, child and adolescent mental health services, public health or school nurses, and last but not least the child protection services. It has never been the intention that the adult mental health services were to provide for these children within their services. Furthermore, a relevant question is if the child responsible personnel in the adult mental health services are aware of the services available for children of mentally ill parents in the local communities. They may not know the available alternative for interventions and services in the municipalities, and may therefor profit from collaboration with relevant agencies. This has been shown in a previous study where more than one-third of the child responsible personnel in Norwegian hospitals did not consider it their task to have an overview of services available in community services [3]. Additionally, many local communities also have limited resources and few available interventions or programs for children of mentally ill parents, and the adult mental health services may encounter a lack of support from the municipalities due to lack of competence, knowledge, financial resources and qualified personnel.

## Study limitations

One important limitation of the present study that we only examined attitudes, knowledge, and current work practice based on personnel’s own perceptions. Consequently, all clinics may have designated child responsible personnel, but the staff may not be informed who they are. A result of this could be reduced impact of the effort to support patients’ children. Future studies may also include objective measures, e.g., journal data reporting on family assessments.

Another limitation was the relatively modest response rate of this study, especially at time 3. The number of respondents have dropped significantly from 2010 to 2015, especially child responsible personnel, and this may have compromised the results of wave three. The independent t-tests assumptions of equal variances were violated, and this implies that extra caution must be held when interpreting the results. We believe the interest to participate in 2010 was related to the focus and interest on the topic children of mentally ill parents at this crucial point, as part of the effort to make the legislative changes known in Norwegian hospitals. In 2015 children of mentally ill parents were no longer a “hot topic”, and there are probably other topics that now have a greater focus.

Furthermore, due to the anonymity of the respondents, the samples at pre, post and follow-up tests are not independent. This means that the respondents may have been influenced by being nested within the same department, and this may have biased the findings in the study. In future research personnel should be evaluated individually to track changes in levels of competence and clinical practice.

## Conclusion

In general, the results of this study suggested that the systems change of establishing child responsible personnel within adult mental health services to safeguard children of mentally ill parents could be a tool. The child responsible personnel were considered to have more knowledge about risk factors for families in relation to parental mental illness, and to have higher awareness about the legislation in this area. However, the role of being a child responsible should be further developed and defined. The safeguarding of patients’ children should in some way be related to formal authority and decision making processes in the clinics. As a consequence, we believe that leaders in the clinic should consider appointing the therapists, i.e., the psychologists or the psychiatrists. They are in a significant position to direct the treatment process, and hence to bring up the parenting role with the patient. As such, they will have the information and the authority to act when it is necessary to initiate interventions to safeguard the children of patients. Furthermore, this study showed the importance of an ongoing focus in order to successfully implement a systems change in adult mental health services. Sustainability of new routines will be impossible if there is no attention and resource allocation to the change that was intended in the first place.

## Abbreviations

CR: child responsible
GS: general staff
ES: effect size
